# The Potential of Microbial Fuel Cells for Remediation of Heavy Metals from Soil and Water—Review of Application

**DOI:** 10.3390/microorganisms7120697

**Published:** 2019-12-13

**Authors:** Chaolin Fang, Varenyam Achal

**Affiliations:** 1 Environmental Engineering Program, Guangdong Technion-Israel Institute of Technology, Shantou 515063, China; chaolin.fang@gtiit.edu.cn; 2 Technion-Israel Institute of Technology, Haifa 3200, Israel

**Keywords:** microbial fuel cell, microbial electrolysis cell, heavy metals, wastewater

## Abstract

The global energy crisis and heavy metal pollution are the common problems of the world. It is noted that the microbial fuel cell (MFC) has been developed as a promising technique for sustainable energy production and simultaneously coupled with the remediation of heavy metals from water and soil. This paper reviewed the performances of MFCs for heavy metal removal from soil and water. Electrochemical and microbial biocatalytic reactions synergistically resulted in power generation and the high removal efficiencies of several heavy metals in wastewater, such as copper, hexavalent chromium, mercury, silver, thallium. The coupling system of MFCs and microbial electrolysis cells (MECs) successfully reduced cadmium and lead without external energy input. Moreover, the effects of pH and electrode materials on the MFCs in water were discussed. In addition, the remediation of heavy metal-contaminated soil by MFCs were summarized, noting that plant-MFC performed very well in the heavy metal removal.

## 1. Introduction

Fossil fuels are the most widely used but exhaustible energy sources and it is estimated that oil, gas, coal, or uranium will be depleted in 55–75 years [1]. On the other hand, the continued use of fossil fuels has brought a series of environmental issues, such as enormous carbon dioxide emission and thermal pollution. In the context of the global energy crisis and environmental issues, researchers are exploring solar, wind, tidal, geothermal, biomass energy, and other biofuels as alternative renewable energy sources. Such resources are increasing dramatically in recent years. Notably, microbial fuel cells (MFCs) are considered as an alternative strategy because of their excellent abilities to produce electricity and remove pollutants simultaneously. Dating back to 1911, the potential of microbes in generating electricity was revealed by Potter’s work that a voltage of 0.3–0.5 V was obtained when a platinum electrode was inserted into a liquid suspension of yeast and *Escherichia coli* using a glucose medium [2]. This discovery was the advent of MFCs research. Nevertheless, early attention to MFCs was in the 1950s as research communities were looking for a new technique, which could convert human waste to electricity timely during space flights [3]. Until the 1980s, redox-active mediators were widely used in MFCs, and it significantly improved the power density output of MFCs. At this point, the feasibility of MFCs as small power supplies increased and more researchers got interested in the research and development of MFCs in regard to alternative renewable energy systems.

**Figure 1:**
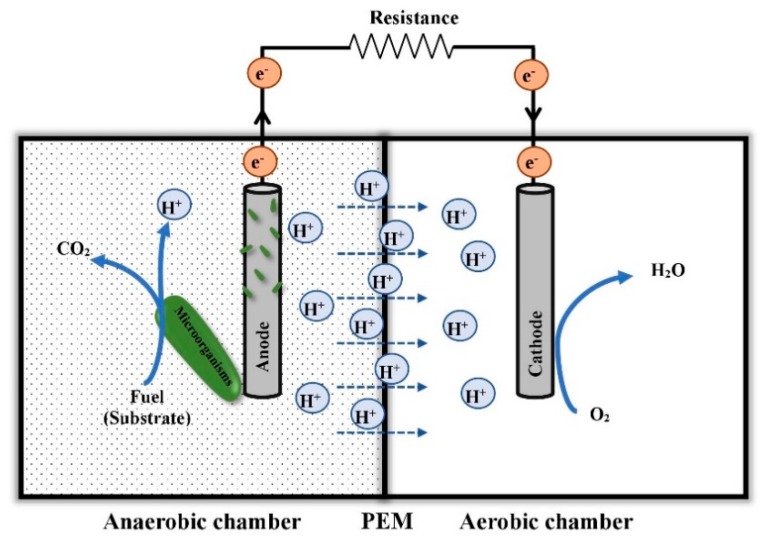
Schematic of a typical two-chamber microbial fuel cell.

In simpler words, MFCs are devices that use exoelectrogenic microorganisms as biocatalysts and then convert chemical energy to electrical energy directly via substrate oxidation [4,5]. A typical electrode reaction using acetate as a substrate [6] and a two-chamber MFC setup (Figure 1) are shown below:

(1) $${\mathrm{Anodic}\;\mathrm{reaction}:\;\mathrm{CH}}_{3}{\mathrm{COO}}^{-}{+2H}_{2}O\;\overset{\mathrm{microbes}}{\to }{2\mathrm{CO}}_{2}{+7H}^{+}{+8e}^{-}$$

(2) $${\mathrm{Cathodic}\;\mathrm{reaction}:\;O}_{2}{+4e}^{-}{+4H}^{+}\to {2H}_{2}O.$$

In MFCs, microorganisms oxidize organic substrates in the anode chamber under anaerobic condition, resulting in the production of protons and release of electrons, and electrons flow from the anode to the cathode through an external circuit. These electrons combine with protons and an electron acceptor (mainly oxygen) on the cathode surface to produce water. Plus, the anode chamber and the cathode chamber get separated by a proton exchange membrane (PEM) that allows protons to transfer from anode to cathode and prevents oxygen diffusion to the anode chamber [7]. The developments and advancements of MFCs have been made to improve electricity generation over the years, for instance, enhancing bacterial electron transfer toward the MFCs anode [8,9], optimizing electrode materials [10,11], and developing different MFCs configurations [12,13].

More remarkably, MFCs systems have been used to accomplish wastewater treatment/bioremediation along with power generation [14,15,16,17]. Exoelectrogenic microorganisms in MFCs could utilize nearly any source of biodegradable organic or inorganic matter that does not directly require oxygen as a part of the degradation process in wastewater for electrical power generation [18]. When MFCs are implemented in wastewater treatment, there are some obvious advantages, including: (i) serving as energy neutral wastewater treatment, while conventional aerobic treatments consume large amounts of electrical energy for aeration [19,20]; (ii) generating less excess activated sludge compared to anaerobic digesters [21]. It is worth noting that the attention for the application of MFCs regarding the biological reduction of heavy metals from wastewater and soil has increased in recent years.

Although it is well known that heavy metals are natural components of the ecosystem [22], they are also the most dangerous species among environmental pollutants [23]. Copper (Cu), chromium (Cr), cadmium (Cd), lead (Pb), and mercury (Hg), among other heavy metals ions, would accumulate in the body through the food chain rather than degrade into the harmless products [24] and may induce many adverse effects on living organisms due to their high toxicity and carcinogenicity even at trace levels [25,26]. Currently the large amount of heavy metals in water and soil are released from anthropogenic sources, such as mining, metallurgy, electroplating, electrolysis, batteries, tanneries, pesticide, and fertilizer. The World Health Organization (WHO) set the guideline value of heavy metals for drinking water, for instance, 2 mg/L Cu, 0.05 mg/L Cr, 0.003 mg/L Cd, 0.01 mg/L Pb, 0.006 mg/L Hg, and 0.01 mg/L As [27]. However, the maximum contaminant levels for these heavy metal are often far exceeded in many water sources because of various wastewater sources [28]. According to the official data, although the overall level of heavy metal emissions in China tended to decrease over the past ten years, the emission of heavy metals from wastewater remains huge. In 2017 alone, high amounts of metals were released including Pb of 38,342.2 kg, Cd of 7126.9 kg, As of 34,317.0 kg, Cr(IV) of 27,711.5 kg, and Hg of 880.2 kg [29]. At the same time, heavy metal-contaminated soil also has been reported in China and many other countries [30,31,32].

At present, heavy metal pollutions are regarded as the most serious environmental problems and heavy metal removal from wastewater and soil has been extensively investigated and studied. MFCs could attract so much attention from worldwide researchers as they are satisfactorily functioning on the remediation of heavy metal pollution in water and soil together with power generation. In this work, the objectives were to review the application of MFCs on the biological reduction of heavy metals from wastewater and soil. Meanwhile, some factors that affect the MFCs’ performances were discussed to provide more information for future studies. Furthermore, the outlooks were raised at the end of this paper.

## 2. Effect of Factors on Heavy Metal Reduction from Wastewater in MFCs

### 2.1. Effects of pH

The presence of PEM is a transport barrier for the protons to cross membrane diffusion. At the earlier stage of MFC, the production rate of protons in the anode exceeds its consumption rate in the cathode chambers. Thus, there is a pH shift and finally it does reach a dynamic equilibrium [6]. The pH of the electrolyte is crucial to the MFCs’ stability and the bioelectrochemical performance and metal recovery of MFCs in wastewater. Generally, a neutral pH condition in the anodic chamber is preferable for the growth of exoelectrogenic microorganisms [33], while many metals’ reduction reactions in the cathodic chamber are strongly dependent on low pH [34,35]. 

One typical Cr(VI) reduction in MFC is presented in Equation (3) and the low pH could positively affect the metal’s recovery. Higher H^+^ serving as enough reactant participated in the reduction reaction [36]. According to Nernst’s equation (Equation (4)) and the definition of cell potential (E_cell_) (Equation (5)), E_cell_ would be increased by increasing H^+^ and initial Cr^6+^ concentration. Furthermore, the maximum power density increment might be due to a decrease in internal resistance resulting from the increase of ionic strength [37]. In the literature, the internal resistance was 300 Ω at 50 ppm Cr^6+^ and then decreased to 100 Ω at 500 ppm Cr^6+^ [38].

(3) $${\mathrm{Cr}}_{2}O_{7}^{2-}{+14H}^{+}{+6e}^{-}\to {2\mathrm{Cr}}^{3+}{+7H}_{2}O\;\left(E^{0}\;=\;1.33\;V\right)$$

(4) $$E_{\mathrm{cat}}{\;=\;E}_{\mathrm{cat}}^{0}-\frac{\mathrm{RT}}{\mathrm{nF}}\ln\frac{{\left[{\mathrm{Cr}}^{3+}\right]}^{2}}{\left[{\mathrm{Cr}}_{2}O_{7}^{2-}\right]{\left[H^{+}\right]}^{14}}$$

(5) $$E_{\mathrm{cell}}{\;=\;E}_{\mathrm{cat}}{-E}_{\mathrm{an}}$$

where E^0^_cat_ is the standard cathode potential (1.33 V vs. Standard Hydrogen Electrode, SHE; H^+^ = 1 M, pH = 0); The thermodynamic anode potential (E_an_) at pH 7 and 298.15 K is a constant; R is the ideal gas constant (8.314 J/mol K); T is the temperature (K); n is the number of electrons exchanged; F is the Faraday’s constant (96,485 C/mol); [H^+^], [Cr^3+^], and [Cr_2_O_7_^2−^] are the concentrations of the three types of ions in solution.

Cu^2+^ may precipitate as CuO, Cu_2_O, or other forms not available for reduction at pH 4.5 or higher and low pH is necessary for the copper reduction in a cathode chamber [39]. During the cathodic reduction of Cu^2+^ in MFC, two products of Cu or Cu_2_O could be formed on the cathode in MFC (Equations (6) and (7)). H^+^ has no effect of on Cu^2+^ reduction to pure Cu based on Equation (6). However, under a low-pH environment, the increase of H^+^ concentration leads to the increase of E_cat_ (Cu_2_O/Cu) and the decrease of E_cat_ (Cu^2+^/Cu_2_O) demonstrated in the following Nernst equation (Equations (9) and (10)) [40]. As a result, Cu^2+^ to Cu would be more favorable than Cu^2+^ to Cu_2_O and more Cu_2_O could be further reduced to Cu (Equation (8)).

(6) $${\;\mathrm{Cu}}^{2+}{+2e}^{-}\to {\mathrm{Cu}\;(E}^{0}\;=\;0.337V)$$

(7) $${2\mathrm{Cu}}^{2+}{+H}_{2}{O+2e}^{-}\to {\mathrm{Cu}}_{2}{O+2H}^{+}\left(E^{0}\;=\;0.207V\right)$$

(8) $${\mathrm{Cu}}_{2}{O+2e}^{-}{+2H}^{+}\to {2\mathrm{Cu}+H}_{2}{O\;(E}^{0}\;=\;0.059V)$$

(9) $$E_{\mathrm{cat}}\left({\mathrm{Cu}}^{2+}{/\mathrm{Cu}}_{2}O\right){\;=\;E}_{\mathrm{cat}}^{0}\left({\mathrm{Cu}}^{2+}{/\mathrm{Cu}}_{2}O\right)-\frac{\mathrm{RT}}{\mathrm{nF}}\ln\frac{{\left[H^{+}\right]}^{2}}{{\left[{\mathrm{Cu}}^{2+}\right]}^{2}}$$

(10) $$E_{\mathrm{cat}}\left({\mathrm{Cu}}_{2}O/\mathrm{Cu}\right){\;=\;E}_{\mathrm{cat}}^{0}\left({\mathrm{Cu}}_{2}O/\mathrm{Cu}\right)-\frac{\mathrm{RT}}{\mathrm{nF}}\ln\frac{1}{{\left[H^{+}\right]}^{2}}$$

In another study, the maximum Hg^2+^ removal was realized at pH 2 together with the maximum power generation of 318.7 mW/m^2^ higher than 4.21 ± 0.34, 4.84 ± 0.00, and 5.25 ± 0.36 mg/L for an initial pH of 3, 4, and 4.8, respectively. H^+^ concentration also has no effect of the reduction of Hg^2+^ and its reduced product of Hg_2_^2+^ according to reduction equations (Equations (11)–(13)), but H^+^ enhanced the removal of Hg^2+^ by chemical mechanism [41]. Hg_2_^2+^ was precipitated in the presence of Cl^−^ (Equation (14)) and Hg_2_Cl_2_ dissolved at low pH to generate higher Hg^2+^. Subsequently, Hg_2_^2+^ was further reduced to Hg(0) and the mercury concentration decreased in effluent (Equation (15)). In addition, the maximum power density increased from 8.9 to 318.7 mW/m^2^ with a decrease in pH value from 4.8 to 2. H^+^ does not determine the standard potential of Hg^2+^ or Hg_2_^2+^, according to the Nernst equation. Herein, the effect of pH on the maximum power densities should be due to the decreased internal resistance caused by the increase of ionic strength.

(11) $${2\mathrm{Hg}}^{2+}{+2e}^{-}\to {\mathrm{Hg}}_{2}^{2+}{\;(E}^{0}\;=\;0.911\;V)$$

(12) $${\mathrm{Hg}}_{2}^{2+}{+2e}^{-}\to 2\mathrm{Hg}\;\left(E^{0}=\;0.796\;V\right)$$

(13) $$H^{2+}{+2e}^{-}\to \mathrm{Hg}\;\left(E^{0}\;=\;0.851\;V\right)$$

(14) $${\mathrm{Hg}}_{2}^{2+}{+2\mathrm{Cl}}^{-}\to {\mathrm{Hg}}_{2}{\mathrm{Cl}}_{2}$$

(15) $${\mathrm{Hg}}_{2}{\mathrm{Cl}}_{2}{+2e}^{-}\to {2\mathrm{Hg}+2\mathrm{Cl}}^{-}\left(E^{0}\;=\;0.268\;V\right)$$

### 2.2. Effects of Cathode Materials

Clearly, the cathode material is a very important factor for the MFC system, and it affects the performance of MFCs. Typically, a good cathode material should have the following properties: excellent electrical conductivity, large surface area, good stability, and economic. The commonly used carbon-based cathode materials contain graphite (e.g., graphite foil, graphite plate, graphite felt, graphite rod, etc.) and plain carbon (e.g., carbon cloth, carbon paper, carbon brush, carbon felt, carbon fiber, carbon rod, etc.). Metal-based cathodic materials, such as stainless steel mesh, nickel foam, titanium sheet, also have been used as cathode electrodes in MFCs [42,43] and they have advantages of higher electrical conductivity, avid facilitation of microbial adhesion, and durability [5]. But obviously, these metal-based cathodic materials are not cheap and increase the cost of the MFCs.

Activation losses, ohmic losses, and mass transport losses in voltage reduce the real voltage output (V_op_) and affect MFC performance. When the thermodynamically predicted voltage of an MFC subtracts the voltage losses of each compartment, V_op_ can be determined as follows [44]:

(16) $$V_{\mathrm{op}}{=E}_{\mathrm{cell}}-\left[{\left(\eta_{\mathrm{act}}{+\eta }_{\mathrm{ohmic}}{+\eta }_{\mathrm{conc}}\right)}_{\mathrm{cat}}+{\left(\eta_{\mathrm{act}}{+\eta }_{\mathrm{ohmic}}{+\eta }_{\mathrm{conc}}\right)}_{\mathrm{an}}\right]$$

where E_cell_ is the thermodynamic cell voltage, η_act_ is the activation loss due to reaction kinetics, η_ohmic_ is the ohmic loss from ionic and electronic resistances, and η_conc_ is the concentration loss due to mass transport limitations.

The cathode internal resistance is an important limiting factor for the power output of MFCs, which is further divided into charge transfer resistance of the electrodes, ohmic resistance, and diffusion resistance [45]. Electrochemical impedance spectroscopy (EIS) can be used to determine the individual components of the internal resistance. The cathodic ohmic includes the resistance from the electrode, member, electrolytes, and interconnections in the cathode and it plays a very minor role in comparison with charge transfer resistance [46]. Moreover, the cathodic ohmic could be reduced by changing the cell design and shortening the electrode distance [47]. Liu et al. [37] reported that the a decrease of the distance between the anode and cathode that was from 4 to 2 cm resulted in an increase of the power density from 720 to 1210 mW/m^2^. The charge transfer resistance is related to the activation energy of the cathode. A smaller charge transfer resistance of the cathode indicates a higher kinetic driving force and a lower hindrance to charge transport [48]. Sometimes, cathode materials are coated with catalysts (e.g., Pt, MnO_2_) to increase the kinetics of oxygen reduction at the electrode surface and reduce the cathodic reaction activation energy [44,49]. In a Cr(VI) reduction experiment via MFC system using carbon cloth, carbon brush, and carbon felt as cathode materials [50], the ohmic resistance of three electrode materials were 5.6, 4.8, and 4.3 Ω, respectively and the charge transfer resistance of three electrode materials were 7.52, 73.86, and 113.66 Ω, respectively. In terms of Cr(VI) removal efficiency, results showed a higher rate constant value of 0.1567 per hour (0.1567 h^−1^) in MFC with carbon cloth cathode than that of carbon brush cathode (0.1064 h^−1^) and carbon felt cathode (0.0719 h^−1^). Three MFC systems achieved the complete Cr(VI) removal in 72, 300, and 444 h, respectively. At the same time, the maximum power density MFC with carbon cloth electrode was 1221.91 mW/m^2^ and it was 2.69 folds and 8.49 folds higher than that of carbon brush electrode and carbon felt electrode, respectively.

## 3. MFCs Performances on Heavy Metal Removal in Wastewater

To date, various physical, chemical, and biological technologies have been developed for the removal of heavy metals from wastewater and soil, such as chemical precipitation, ion exchange, membrane filtration, biosorption, bioremediation, etc. [25,28,51]. However, there are some technical or economic constraints that always restrict the application of these techniques, including high energy input, excessive chemicals consumption, and substantial toxic waste sludge [35,52,53]. On the other hand, MFCs are cost-effective and environmentally friendly technologies, and they can reduce heavy metals from wastewater and soil with high removal efficiency and electricity generation [54].

MFCs are constructed to remove heavy metals through the cathodic reduction reaction, while organic substrates are oxidized and serve as the carbon and electron donor at the anode [34]. In addition, previous studies found that biosorption and precipitation reactions (sulfides, hydroxides) also made significant contributions on the removal of heavy metals in MFC systems [55,56,57]. A summary of some applications of MFCs on heavy metal removal in wastewater is presented in Table 1 and the part of metal reductions is described in detail.

### 3.1. Copper Removal

A membrane-less single-chamber MFC was constructed for the copper removal. Such an MFC system can help reduce the MFCs cost from the membrane for the long-term wastewater treatment [58]. Results showed that copper was removed efficiently with a removal efficiency of 98.3% in 28 h at the tolerable Cu^2+^ concentration of 12.5 mg/L and the product of CuO and Cu_2_O was deposited on the biocathode. The maximum power density was 10.2 W/m^3^. The main functional exoelectrogen and tolerant copper microorganisms were from the phylum Proteobacteria and the genus of *Acinetobacter* and *Geobacter* with *Geobacter metallireducens* and *Geobacter sulfurreducens* as prime species. Meanwhile, it was observed that Cu^2+^ concentration was negatively associated with voltage and maximum power density that could be due to higher Cu^2+^ concentration inhibiting the activity of microorganisms. 

A bipolar membrane was applied to maintain the pH difference between the anode and cathode chambers [59]. Under anaerobic and aerobic conditions, the metallurgical MFC demonstrated excellent performances on copper reduction in copper-containing waste streams, indicating that the maximum copper removal efficiency was calculated to be 99.88% and 99.95% in seven days, respectively. MFC system also improved the maximum power density of 0.43 to 0.80 W/m^2^. In this process, scanning electron microscopy and X-ray powder diffraction confirmed that the product of copper removal was pure copper crystals, not CuO or Cu_2_O [39].

### 3.2. Hexavalent Chromium Removal

In general, hexavalent chromium Cr(VI) and trivalent chromium Cr(III) are main valence states of chromium in the natural environment. Cr(VI) is water soluble with high toxicity in the full pH range, while Cr(III), being less mobile, is less toxic and tends to form Cr(OH)_3_ precipitates. The reduction of Cr(VI) to Cr(III) is a common remediation pathway of wastewater containing Cr(VI) [34]. Li et al. [50] operated a two-chamber MFC to reduce Cr(VI) to non-toxic Cr(III) using three different cathode materials—the carbon cloth, carbon brush, and carbon felt. Results showed that 80 mg/L Cr(VI) was completely removed by MFC with carbon cloth cathode in 72 h at an optimized pH of 2 while only 33.45% and 12.72% of Cr(VI) removal efficiency were obtained using carbon brush and carbon felt as cathode materials, respectively. The MFC maximum power density with carbon cloth electrode was 1221.91 mW/m^2^, which was 2.69 folds and 8.49 folds higher than that of carbon brush electrode and carbon felt electrode, respectively.

It is a common case that one practical wastewater contains multiple metals, for example, Cr(VI) and vanadium V(V) are common pollutants in vanadium-containing wastewater. Herein, when MFC was applied for the remediation of such wastewater, V(V) and Cr(VI) acted as two different electron acceptors in double-chamber MFC [60]. As a result, the V(V) and Cr(VI) reduction efficiencies were 67.9% ± 3.1% and 75.4% ± 1.9% in 240 h, respectively, with a maximum power density of 970.2 ± 20.6 mW/m^2^. The product of Cr(VI) reduction mainly was Cr(III) in the form of hydroxide deposited on the cathode and vanadium was precipitated or soluble depending on the pH of exhausted catholyte.

### 3.3. Cadmium (Cd) Removal

The standard redox potential of the Cd(II)/Cd(0) is −0.40 V and it is difficult to make its reduction into elemental metal by a single MFC because MFCs cannot generate a sufficient voltage and power. Microbial electrolysis cells (MECs) are regarded as potential bio-electrochemical technologies to reduce heavy metals with external power supply request and have been used to reduce Cd (II) [61]. Choi et al. [62] studied that Cd (II)-MEC coupled with Cr(VI)-MFC to recover Cd (II) without external energy input. Consequently, Cr(VI)-MFC could successfully complement the insufficient voltage and power needed during the Cd(II) reduction in Cd(II)-MEC system. After 60 h reaction, the removal efficiencies of 200 ppm Cr(VI) and 50 ppm Cd(II) were 13.95% ± 0.73% and 93.43% ± 0.17%, respectively. The maximum output power (22.5 Wm^2^) of Cr(VI)-MFC was 11.3 times higher than the highest power density supplied to Cd(II)-MFC (2.0 Wm^2^). Plus Pb(II)-MEC connected with Cr(VI)-MFC were also designed to simultaneously reduce Cr(VI) and Pb(II) successfully without external energy input [42].

The effects of Cd on the power generation of a single-chamber air-cathode MFC and Cd removal in MFC were investigated by Abourached et al. [55]. Firstly, the Cd maximum tolerable concentration of the electrochemically active microorganisms was 200 mM in MFC. The decreased rate of MFC voltage output caused by the gradual increase of Cd concentrations with an 100 mM increment was slower than that of MFCs, which were fed with 300 mM Cd medium solution directly. Notably, the high power generation of 3.6 W/m^2^ was achieved simultaneously with a high Cd removal efficiency of 90%. However, the probable product of Cd removal was CdS and the high removal efficiency of Cdwas realized mainly through biosorption and sulfide precipitation rather than through cathodic reduction.

### 3.4. Mercury (Hg) Removal

Wang et al. [41] investigated the effects of the pH and initial Hg^2+^ concentration on the performance of a two-chamber MFC for Hg^2+^ removal and power generation. After a 5 h reaction, the initial 50 mg/L Hg^2+^ was deceased to 3.08 ± 0.07, 4.21 ± 0.34, 4.84 ± 0.00, and 5.25 ± 0.36 mg/L at pH 2, 3, 4, and 4.8, respectively. In addition, higher removal rate was realized with higher initial Hg^2+^ concentration in 4 h. The maximum power generation was obtained at pH 2 with the initial Hg^2+^ concentration of 100 mg/L, about 433.1 mW/m^2^ in comparison to that of lower initial Hg^2+^ concentration (25 and 50 mg/L).

### 3.5. Silver (Ag) Removal

Tao et al. [63] confirmed that the recovery of silver through the cathodic reduction in MFCs was related to metal forms in the terms of Ag(I) ions and Ag(I) thiosulfate complex [AgS_2_O_3_]^−^. Firstly, the increased pH (from 2 to 6.6) had less impact on the reduction of Ag^+^, but higher pH tended to accelerate the reduction of [AgS_2_O_3_]^−^. The reduction rate of Ag^+^ ions was higher than that of [AgS_2_O_3_]^−^ at pH 4.0. For Ag^+^ of 110 mg/L from the simulated photographic wastewater. In addition, the Ag^+^ concentration decreased faster in the electrolysis reactors than in MFCs before reaching 20 mg/L. After that, Ag(I) declined continuously and the final removal efficiency in MFCs was above 95% versus 80% in the electrolysis reactors.

### 3.6. Thallium (Tl) Removal

The redox potential of the pair Tl(III)/Tl(I) (*E*^0^ = −1.26 V) indicates that the oxidation of Tl(I) to Tl(III) is relatively easier to achieve. Unlike other cathodic reduction of heavy metals in MFCs, Tl(I) was oxidized to Tl(III) in the anode and then precipitated as Tl(OH)_3_ which would be easier to be removed from aqueous solution. A single-chamber air-cathode MFC was constructed to support spontaneous Tl(I) oxidation with electricity generation [64]. Result showed that the Tl(I) removal efficiency was over 67.2% ± 2.3% in 72 h with the initial Tl(I) concentration of 100 μg/L, and the maximum power density of 457.8 ± 15.2 mW/m^2^ was obtained.

On practical aspects, MFCs were applied for the remediation of various heavy metals by bioelectrocatalysis as metal ions can be reduced and deposited by microbes. Almost all silver (Ag) and vanadium (V) was removed using two chamber MFCs, when concentrations were 50 and 200 mg/L, respectively [65,66]. Microbial-assisted Cr(VI) reduction was reported in a two-chamber microbial fuel cell where *Trichococcus pasteurii* and *Pseudomonas aeruginosa* played a major role [67]. A very high concentration of vanadium (V) with 300 mg/L NaVO3 into the anode chamber with *Rhodoferax ferrireducens* was treated that resulted in a reduction ratio of NaVO_3_ of 75.8% [68].

In one of studies, it was found that 1.6 g Ag could be deposited on cathode with over 99.9% silver ions removed from the catholyte in the treatment of acetate wastewater [69]. About total of 50 and 200 mg Se/L selenite was removed in 48 and 72 h, respectively using MFCs [70]. While treating 1000 mg/L Cu(II)-containing wastewater, about 98% of cathodic Cu(II) was removed in a dual-chamber MFC in 288 h [71]. The 96% cathodic reduction of Cu(II) was 96% for an initial concentration of 350 mg Cu per L in 12 h [72]. A total of eight heavy metals including Se, Ba, Sr, Zn, Mo, Cu, Cr, and Pb were removed with as high as 97.8% of selenium during the treatment of oil sand tailings [73].

Although MFCs were applied to treat most of the heavy metals, the majority of researchers so far focused on Cr removal that could be due to its high redox potential. Still, there exists limitation due to obstacles in the system architecture of MFCs in some cases and process scale-up in heavy metal treatment. The environmental conditions (sun, wind, rain, temperature, etc.), excess biofilm growth on electrodes, and indigenous microbial community could pose additional problems to run MFCs practically, especially on-site or in field studies.

## 4. Biological Reduction of Heavy Metals from Soil in MFCs

The feasibility of MFCs in remediating heavy metal-contaminated soil was investigated in some studies; however, to date, the studies of soil MFCs for heavy metals are relatively few. A summary of some soil MFC applications on heavy metal removal is presented in Table 2. The remediation of heavy metals in soil driven by MFCs is better described as the metal’s migration from the anode to the cathode. As a consequence, after remediation, the heavy metal concentration increases in the cathode regions and decreases in the anode regions.

A soil MFC was constructed to produce electricity and remediate Cu-contaminated soil by Wang et al. [74]. Results showed that the Cu migration process was enhanced by the electrical field, not diffusion or leaching, and the time and space distribution of water soluble and total copper changed obviously with the increased metal concentration gradient from the anode to the cathode. Meanwhile, the soil pH changed significantly compared to control group and the pH gap between the cathode and the anode reached 2.25 units. The similar pH variation and the migration process of Pb and Cd in soil were also reported in other study [75]. In addition, soil is a more complex medium than water, and soil properties affect the remediation efficiency of metal-contaminated soil using MFCs [76,77]. 

The remediation of two Cr(VI)-contaminated soils: red clay soil and fluvo-aquic soil, using MFCs with two different external resistances were investigated. After remediation for 16 days, the Cr(VI) removal efficiency in the fluvo-aquic soil was 99.1% with 100 Ω and 64.3% with 1000 Ω, much higher than those in the red clay soil (62.7% with 100 Ω and 50.4% with 1000 Ω). MFCs can serve as a potential and sustainable approach to remediate heavy metal-contaminated soil, but as shown in Table 2, the metals removal efficiencies in soil by a single MFC are not high. Thus, the combination of soil MFCs and other methods were developed to improve heavy metal removal efficiency, such as plant-microbial fuel cell (PMFC) [16,78].

The coupling of plants to soil MFC is a clever pathway that can convert solar energy into bioelectricity based on a unique plant–microbe relationship, and it is a promising modification of MFC to remediate the metal-contaminated soil in situ [79]. PMFC can utilize root exudates and other organics as substrates to generate electrons with the help of electrochemically active microorganisms in the rhizosphere of a plant. PMFC was applied for the removal of Cr(VI) successfully and the Cr(VI) removal efficiency of 99% was realized [16]. Meanwhile, the maximum current density of the PMFC reached 55 mA/m^2^ at an initial Cr(VI) concentration of 19 mg/L, which was approximately two-times higher than that at an initial Cr(VI) concentration of 9 mg/L. The high Cr(VI) removal efficiency was attributed to plant uptake, bioelectrochemical reduction, direct reduction by Cr-reducing microorganisms, and adsorption by electrodes in PMFC. A large proportion of the total Cr(VI) was removed by bioelectrochemical reduction and plant uptake with an approximate share of 57.1% and 29.4%, respectively. In another study, *Pennisetum alopecuroides* and *Phragmites communis* were selected as wetland plants for the PMFC experiment of the Cr(VI) removal in soil. Result showed that the Cr(VI) removal efficiency reached 99% in all PMFCs systems. The daily average voltage value of PMFC systems of *P. alopecuroides* in closed circuit using the graphite carbon felt electrodes could reach 469.21 mV. In sum, MFCs and their modification to PMFCs are promising techniques for heavy metal-contaminated soil remediation. Moreover, the performances of PMFCs were enhanced significantly compared to that of the traditional MFCs in soil, especially for the high metals removal.

## 5. Conclusions

In conclusion, MFC is a promising technology for simultaneous pollutant reduction and electricity generation. The cathodic reduction coupled to organic substrate oxidation has successfully removed several heavy metals from wastewater and soil. In addition to combining biological technology with electrochemical technology, the unique advantages of MFCs are due to simple operation and easy construction resulting not only in heavy metal removal but also in metal recovery for reuse and recycling. However, the current MFCs are not capable of competing with and replacing the conventional energy generation approach based on fossil fuels and there are still many challenges for their future application. For this, research toward improving power generation, reducing the cost, and long-term operation of MFCs need to be intensely explored.

## Figures and Tables

**Table 1:** Microbial fuel cells (MFCs) for the removal of heavy metals from wastewater.

Metal	MFC Fabrication	Maximum Removal Efficiency	Maximum Power Generation	References
Cu(II)	Single-chamber MFC, Carbon brush for anode, Carbon cloth/Pt coated for cathode	98.3%	10.2 W/m^3^	[40,58]
Cu(II)	Two-chamber MFC, Graphite plate for anode, Graphite foil for cathode	99.88% (Anaerobic) 99.95% (Aerobic)	0.43 W/m^2^ (Anaerobic) 0.80 W/m^2^ (Aerobic)	[39]
Cr(VI)	Two-chamber MFC, Carbon felt for anode, Carbon cloth^(a)^/Carbon brush^(b)^/ Carbon felt^(c)^ for cathode	100%^(a)^ 33.45%^(b)^ 12.72%^(c)^	1221.91 mW/m^2^	[50]
Cr(VI) and V(V)	Two-chamber MFC, Carbon fiber felt for anode; Carbon fiber felt for cathode	75.4% ± 1.9% Cr(VI) 67.9% ± 3.1% V(V)	970.2 ± 20.6 mW/m^2^	[60]
Cd(II)	Single-chamber MFC; Carbon cloth for anode; Carbon cloth/Pt coated for cathode	90%	3.6 W/m^2^	[55]
Cr(VI) and Cd(II)	Two double-chambers MFC–MEC (microbial electrolysis cell), carbon brush and carbon cloth for anode and cathode of both Cr(VI)-MFC and Cd(II)-MEC	13.95% ± 0.73% (for 200 ppm Cr(VI)) 93.43% ± 0.17% (for 50ppm for Cd(II))	22.5 W/m^2^	[62]
Hg(II)	Two-chamber MFC, Graphite felt for anode, Carbon paper for cathode	i.3.08 ± 0.07 mg/L (pH = 2) ii. 99.54% (for 100 mg/L Hg(II))	i. 318.7 mW/m^2^ (pH = 2) ii. 433.1 mW/m^2^ (For 100 mg/L Hg (II))	[41]
Ag(I)	Two-chamber MFC, Carbon brush for anode, Carbon cloth for cathode	99.91% ± 0.00% (for 50 ppm Ag(I))	4.25 W/m^2^ (For Ag^+^ ppm, pH = 7)	[65]
Ag(I)	Two-chamber MFC, Graphite plates for anode, Graphite plate for cathode	i. 0.109 ± 0017 mM ii. 0.025 ± 0.008 mM	109 mW/m^2^ (For acetate COD 500 mg/L)	[63]
Au(III)	Two-chamber MFC Carbon brush for anode, Carbon cloth for cathode	99.88% (for 200 mg/L Au(III))	6.58 W/m^2^ (For 2000 ppm Au(III))	[45]
V(V)	Two-chamber MFC, Carbon fiber felt for anode, Carbon fiber felt for cathode	100%	529 ± 12 mW/m^2^	[66]
Tl(I)	Single-chamber MFC, Carbon felt for anode, Carbon paper/Pt coated for cathode	67.2%	457.8 ± 15.2 mW/m^2^	[64]
Cr(VI) and Pb(II)	Two double-chamber MFC–MEC, Carbon felt anode and carbon cloth cathode for Cr(VI)-MFC; Carbon felt anode and carbon cloth (CC)/ stainless steel mesh (SSM)/ nickel foam (NF) cathode for Pb(II)-MEC	Pb(II) reduction efficiency 100%; Cr(VI) reduction rate 1.72 g/(m^3^·h)	702.86 mW/m^2^ (For NF cathode)	[42]

**Table 2:** MFCs for the removal of heavy metals from soil.

Metal	MFC Fabrication	Reduction Reaction Description	Maximum RemovalEfficiency	Maximum Power Generation	References
Cu(II)	Single-chamber MFC Granular activated carbon for anode and cathode	Cu 600 mg/kg, sodium acetate 6.00 g/L, pH = 7.0, 56 d	N/A	65.77 mW/m^2^	[74]
Pb(II) and Cd(II)	Two-chamber MFC, Graphite granules for anode, Carbon felt for cathode	i. Pb(II) 910 mg/kg, 108 d; ii. Cd(II) 98 mg/kg, 143 d; acetate 1.36 g/L, pH = 6.7	i. 44.1% ii. 31.0%	i. 3.6 mW/cm^2^ ii. 7.5 mW/cm^2^	[75]
Cr(VI)	Two-chamber MFC Carbon felts for anode, Carbon felts for cathode	Cr(VI) 255 mg/kg in red clay soil, pH = 4.80; Cr(VI) 550 mg/kg in the fluvo-aquic soil, pH = 7.04; sodium acetate 2.00 g/L,16 d	99.1% (in fluvo-aquic soil with 100 Ω)	N/A	[76]
Zn(II) andCd(II)	Three-chamber MFC Graphite mat for anode, Graphite mesh/ Pt coated for cathode	Zn(II) 11.6 g/kg, Cd(II) 0.84 g/kg, sodium acetate 2 g/L, 78 d, pH = 3.0	25% (Zn) 18% (Cd)	0.4A/m^2^	[77]
Cr(VI)	Single-chamber plant MFC Graphite granules for carbon, Carbon felt for cathode	Cr(VI) 9.5mg/L and 19 mg/L sodium acetate 1 mM, *Lolium perenne*, 1/2 Hoagland’s solution, greenhouse	99%	55 mA/m^2^	[16]
Cr(VI)	Single-chamber plant MFC Carbon felts or graphite carbon felts for the anode and the cathode	Cr(IV) 500 mg/kg, *Pennisetum alopecuroides*, *Phragmites communis* greenhouse, 96 d	99%	469.21 mV	[78]
